# P-2024. Title: In a Bind: A quality improvement project for reducing quinolone drug-drug interactions with divalent cations

**DOI:** 10.1093/ofid/ofaf695.2188

**Published:** 2026-01-11

**Authors:** Emily Siegrist, Steven Pan, Maria Alkozah, Danh Doan, Bryan White

**Affiliations:** OU Health, Oklahoma City, OK; Hudson College of Public Health, Oklahoma City, Oklahoma; OU Health, Oklahoma City, OK; OU Health, Oklahoma City, OK; University of Oklahoma Medical Center, Oklahoma City, Oklahoma

## Abstract

**Background:**

Ciprofloxacin and levofloxacin have been shown to have a decrease in bioavailability when co-administered with divalent cations such as aluminum, iron, and magnesium. It has been established that drug interactions between quinolones and divalent cations are common in the inpatient setting. The decreased bioavailability of quinolones may drive the development of resistance due to suboptimal plasma concentrations. There is a paucity of data about management of this interaction in modern EMRs. The aim of this study was to evaluate the effectiveness of a quality improvement project in reducing the incidence of quinolone-divalent cation drug-drug interactions.

**Figure 1.**
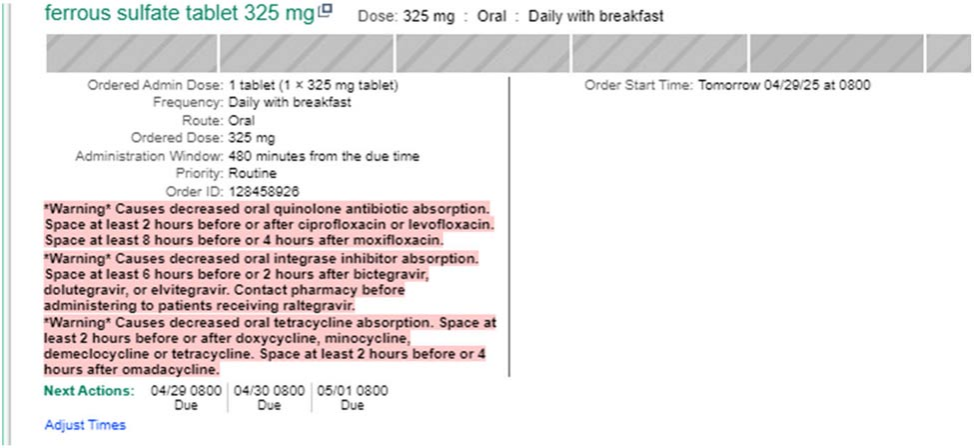
Example of MAR comments when divalent cation and quinolone are both ordered

**Table 1.**
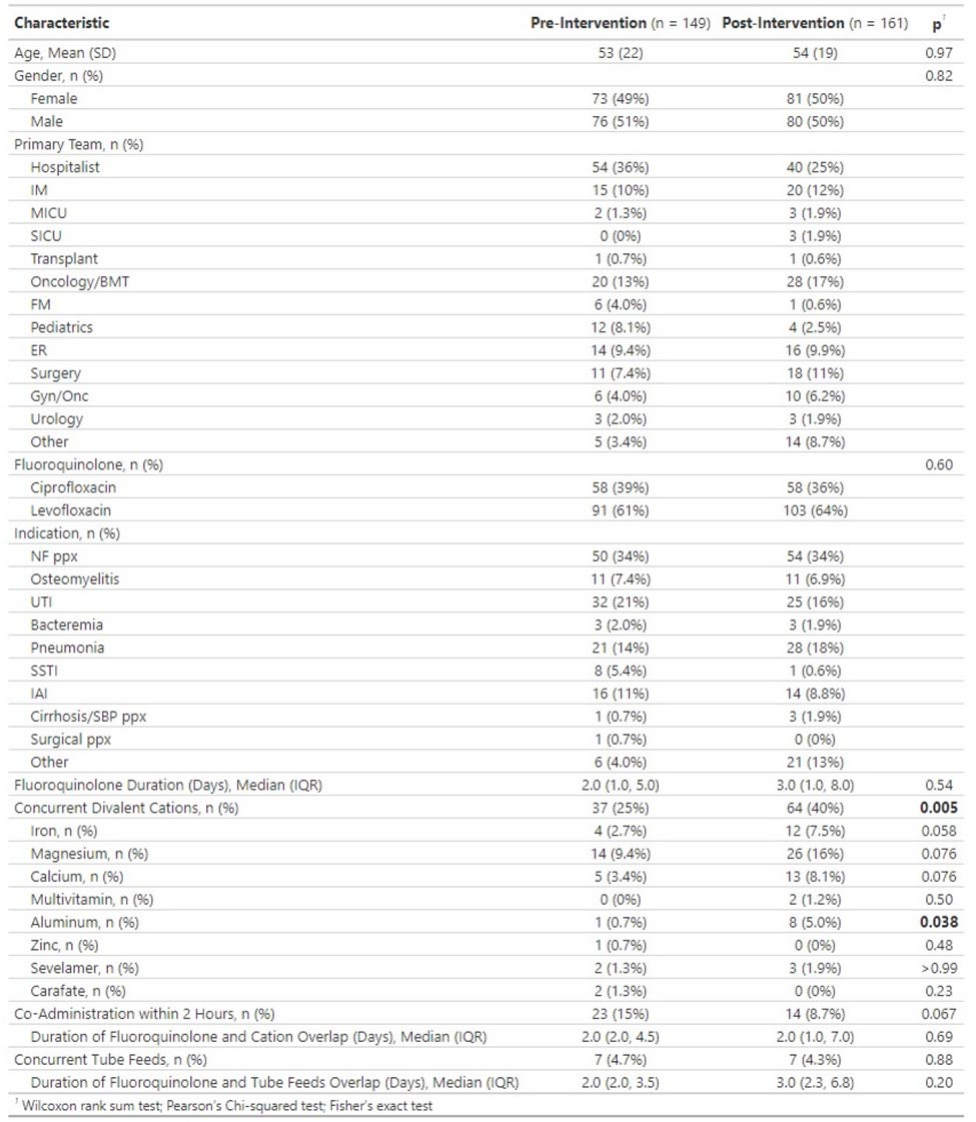
Baseline characteristics and outcomes pre- and post- intervention

**Methods:**

Between November 2024 and January 2024, we rolled out a bundle of interventions targeted towards reducing our divalent cation coadministration within 2 hours of formulary fluoroquinolones, ciprofloxacin and levofloxacin. These interventions included an administration comment visible to nursing on the MAR, nursing education handouts, and alteration of standard time of quinolone administration. Quinolone daily orders were changed to 0600 and twice daily changed to 0600 and 1800. Standard administration time for other orders is 0900 and 2100.

We collected 1 month of quinolone and cation administrations pre-intervention (September 2024) and 1 month post-intervention (February 2025).

**Results:**

We identified 149 patients in the pre-intervention group and 161 patients in the post-intervention group. Divalent cations were co-administered in more patients in the post-intervention cohort (25% vs 40%, *P*=0.005). However, pre-intervention 62% of patients had divalent cations co-administered within 2 hours of the quinolone compared to 22% post-intervention (*P* < 0.001). The median duration of overlap between quinolones and divalent cations was 2 days in both groups. Concurrent tube feeds were similar in both groups (4.7% vs 4.3%, *P* = 0.88) and duration of overlap was the same (2 vs 2 days, *P* = 0.20).

**Conclusion:**

The incidence of multivalent cation - quinolone drug-drug interactions was reduced, but not eliminated, following a bundle of interventions. Incidence of interactions between quinolone antimicrobials and tube feeds did not change.

**Disclosures:**

Bryan White, PharmD, BCPS, BCIDP, Melinta Therapeutics: Honoraria

